# The Effectiveness of Pulsed Dye Laser Combined With CO_2_ Fractional Laser in the Treatment of Port‐Wine Stain in Chinese Patients: A Retrospective Study

**DOI:** 10.1111/jocd.71055

**Published:** 2026-07-24

**Authors:** Jiao Ning, Mengjiao Li, Yang Yang, Guotao Fu, Xiaolei Su, Caiyu Wang, Ping Jian, Jine Zhang, Pu Song, Lin Gao, Bing Li

**Affiliations:** ^1^ Department of Dermatology Xijing Hospital, Air Force Medical University Xi'an Shaanxi China; ^2^ Xi'an Medical University Xi'an Shaanxi China; ^3^ Department of Cardiology Xijing Hospital, Air Force Medical University Xi'an Shaanxi China; ^4^ Department of Dermatology Honghui Hospital, Xi'an Jiaotong University Xi'an Shaanxi China

**Keywords:** CO_2_ fractional laser, port‐wine stains, pulsed dye laser

## Abstract

**Background:**

Port‐wine stain (PWS) is a common type of congenital capillary malformation, often occurring on the face and causing a significant psychological impact on patients. Until now, pulsed dye laser (PDL) remains the criterion standard for PWS treatment, but some patients show poor effectiveness and treatment‐resistance. Fractional laser was previously tried for the treatment in several cases of PWS. However, the effectiveness of its combination with PDL is still unclear.

**Objective:**

This study aims to investigate the effectiveness of PDL combined with CO_2_ fractional laser (CO_2_FL) in treating PWS.

**Methods:**

A retrospective analysis was performed based on the PWS patients who received PDL combined with CO_2_FL treatment in Xijing Hospital, China from January 2014 to December 2024. Clearance rates were recorded to measure clinical effectiveness.

**Results:**

A total of 91 PWS patients were enrolled and analyzed. The age range is from 20 days to 60 years (median age, 11 years), 33 males (36.26%), and 58 females (63.74%). The sites of lesions were predominantly located on the head and face (92.31%), followed by the limbs and trunk (4.40%), and the neck (3.30%). Eighteen patients (19.78%) exhibit nodular hypertrophy type. The results showed that after six sessions of treatments, the average clearance rate was 49.85% ± 22.65%, reaching the plateau. To evaluate the optimal timing of the CO_2_FL combination, we categorized the patients into two groups: those who commenced the combination of PDL and CO_2_FL within the first five sessions, referred to as the early combination group, and those who began the combination after five sessions, designated as the delayed combination group. After eight treatment sessions, compared with the delayed combination group, patients in the early combination group had a higher clearance rate (62.23% ± 23.04% vs. 52.37% ± 22.23%, *p* < 0.05).

**Conclusion:**

The combination of PDL and CO_2_FL is safe and effective for treating PWS. Early combination therapy is associated with better treatment outcomes.

## 1 Introduction

Port‐wine stain (PWS) is a congenital capillary [1, 2, 3] malformation with an incidence rate of 3%–5% [4, 5, 6]. It manifests as red or purplish‐red patches on the skin, predominantly occurring on the face, and significantly impacts patients' appearance and psychology [7, 8]. The pathogenesis of PWS is associated with the abnormal dilation of post‐capillary venules [9]. Currently, pulsed dye laser (PDL) is the first‐line treatment for PWS [10], which selectively photothermally destroys the blood vessels. In East Asian populations, 50%–70% response was achieved after a mean of 5–7 sessions of PDL treatment. 20% patients showed refractoriness or resistance to PDL, especially in cases of blood vessels in the deep dermis or hypertrophic PWS [11, 12]. Additionally, multiple sessions of PDL treatments may cause dyspigmentation, atrophy, and scarring.

Combination therapies with PDL have been proposed to improve clinical effectiveness. Ablative fractional laser (AFL) involves rapid heating and vaporization of skin tissue to the deep dermis in the pattern of fractional photothermy, subsequently stimulating collagen remodeling and angiogenesis to restore normal skin structure [13]. Some previous studies showed that the AFL laser can improve the color and texture of PWS [14, 15]. However, the effectiveness and safety of combination treatment still need further verification. Here, we conduct a retrospective study to analyze the effectiveness of PDL combined with CO_2_FL in treating PWS.

## 2 Materials and Methods

**FIGURE 1:**
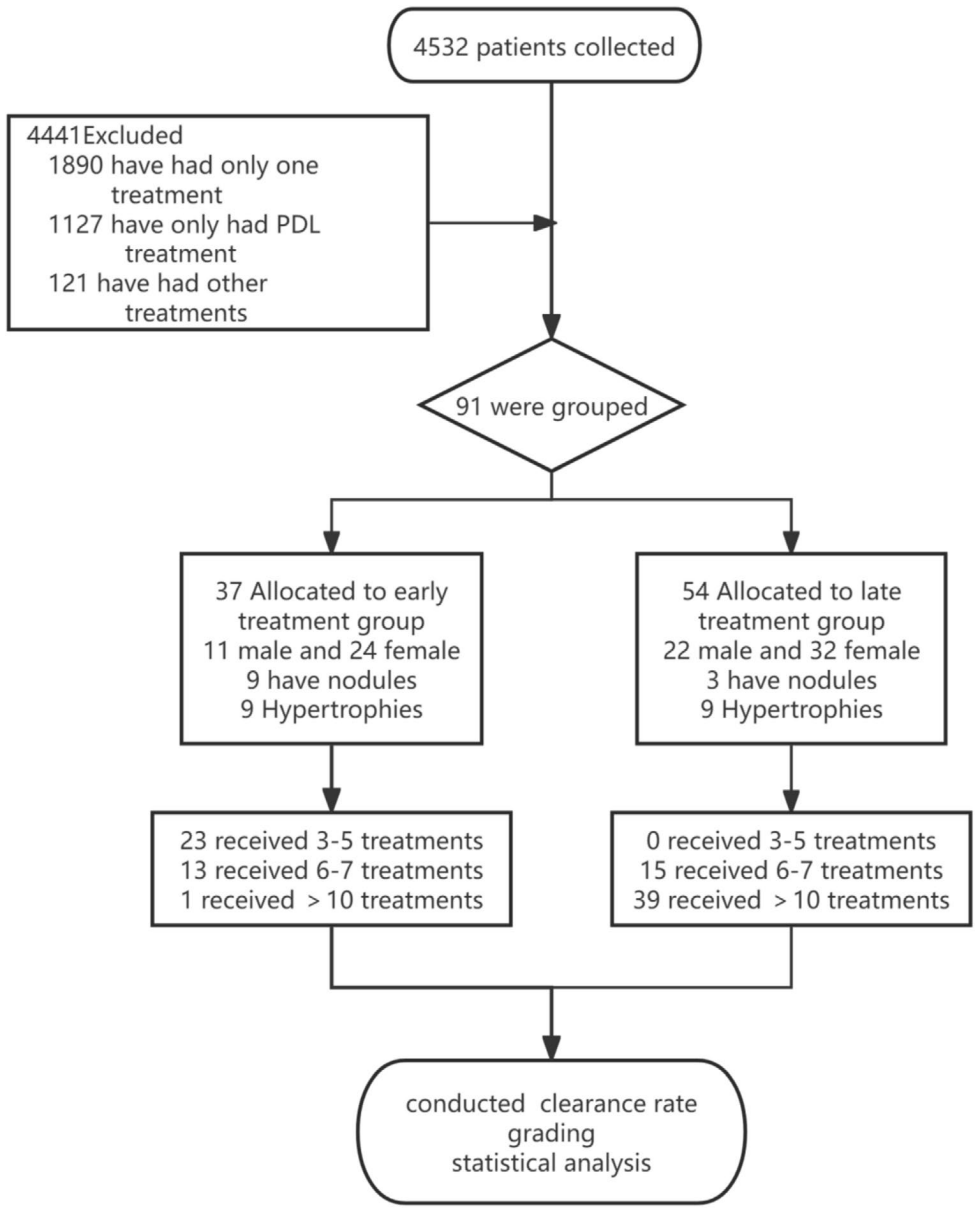
Research flow chart.

This study was a retrospective observational study and was approved by the ethics committee. Informed consent was not required. The overall study design is shown in Figure 1.

### 2.1 Subjects

This cohort included PWS patients who received PDL combined with CO_2_FL treatment in the Department of Dermatology in Xijing Hospital, China from January 2014 to December 2024. Cases with fewer than three sessions of treatment were excluded. The data collection included patient demographics, disease duration, lesion location and type, number of treatment sessions, treatment methods, and photographs with pre‐ and post‐treatment.

### 2.2 Therapy Methods

Pulsed dye laser (PDL) system (Vbeam device, Candela, USA) with a dynamic cooling device (DCD) was used in this study. The parameters were as follows: wavelength of 595 nm, pulse width of 0.45–20 ms, energy density of 8–15 J/cm^2^, spot diameter of 7 mm. The DCD employed ice mist cooling, with a spray duration of 20–100 ms and a delay of 10–100 ms. The combination with CO_2_FL was conducted immediately after PDL treatment. The Acupulse device (LUMENIS, USA) was used for CO_2_FL treatment. The parameters were as follows: wavelength of 10 600 nm, DeepFX mode, energy density of 17.5–30 mJ/cm^2^, a coverage of 5%, an emission frequency of 300 Hz, and a spot diameter of 10 mm without overlap. The detailed treatment parameters were tailored according to factors such as the patient's age, lesion manifestation, and prior treatment outcomes. The treatment was concluded when the lesion's color lightened and appeared purplish‐gray during irradiation. The interval between treatments ranged from 1 to 3 months.

Prior to treatment, patients were informed about the potential normal responses during the procedure, as well as possible adverse reactions that might occur afterward. To safeguard against laser‐induced eye damage, all patients wore metal ocular shields during the treatment. For the younger children, a layer of moist sterile gauze or a disposable eye mask was placed beneath the shields to enhance comfort and ensure effective light‐blocking. Both the operators and accompanying parents were required to wear laser safety goggles throughout the treatment process.

Subsequent to the treatment, ice packs were utilized on the external regions for a period of 5–10 min. Patients were instructed to avoid contact with water and refrain from applying cosmetics for 3 days.

### 2.3 Outcomes

All lesions were captured using a standardized digital camera (EOS 600D, Canon, Japan) under matching lighting conditions at each visit. Comprehensive records were also maintained, documenting patient history, treatment parameters, and any adverse reactions.

The effectiveness of PWS improvement was evaluated using the clearance rate during each appointment. The clearance rate [16] was assessed independently by two skilled dermatologists, blinded to the treatment and to each other's scores, based solely on the photographs. The clearance rate used in the analysis was the mean value of the two evaluators' scores. The clearance rate ranged from 0% to 100%. The evaluation criteria: no effect indicates 0%–25% clearance rate; effective indicates 26%–50% clearance rate; significant indicates 51%–75% clearance rate; cured indicates 76%–100% clearance rate [16].

All Common Terminology Criteria for Adverse Events (CTCAE Versions 5.0) defined events were recorded. Procedural‐related safety endpoints, including hyperpigmentation, atrophy, and scarring, were recorded.

### 2.4 Statistical Methods

Data analysis was performed using WPS and SPSS version 27.0.1.0. Descriptive statistical methods were used for the demographic characteristics of the cases. The Kolmogorov–Smirnov test was applied to continuous variables to assess normality, with *p* > 0.05 indicating normal distribution and *p* < 0.05 indicating non‐normal distribution. If the data were normally distributed, they were presented as mean ± standard deviation (SD); otherwise, they were presented as median (*M*) and interquartile range (IQR), denoted as *M* (Q1, Q3). Categorical variables were expressed as frequencies and percentages. Normally distributed data with homogeneous variance were analyzed using the independent samples *t*‐test, while non‐normally distributed data were analyzed using the Mann–Whitney *U* test. The overall proportion of missing data was 17.82%, with most missing values occurring in the early combination group. Among the early combination group participants, 8 patients completed only three treatment sessions, 10 patients completed four sessions, and five completed five sessions. Twenty patients (86.9%) were very satisfied with the outcomes; thus, we considered that the most missing values were due to achieving satisfactory outcomes in advance. Missing values were handled using multiple imputation [17] using fully conditional specification by SPSS version 27.0.1.0, and five imputed datasets were generated. Analysis of categorical data included chi‐square tests. A significance level of 0.05 was used to determine significant differences in all statistical analyses.

## 3 Results

### 3.1 Demographic Characteristics

In this cohort, we included 91 patients who underwent more than three sessions of PDL combined with CO_2_FL treatment, and their clinical and demographic characteristics are shown in Table 1. There were 33 (36.26%) males and 58 (63.74%) females. The median age was 11 years (range from 20 days to 60 years). According to the Fitzpatrick skin type, there were 9 cases of type II, 22 cases of type III, and 60 cases of type IV. Given the non‐normal distribution of patients' age and number of treatment sessions analyzed by the Kolmogorov–Smirnov normality test, we stratified age into four groups: < 3 years (34.07%), 3–10 years (12.09%), 11–20 years (19.78%), and > 20 years (34.07%). Similarly, five groups were divided based on the number of treatment sessions: 3–5 sessions (23/91, 25.27%), 6–10 sessions (28/91, 30.77%), 11–15 sessions (20/91, 21.98%), 16–20 sessions (12/91, 13.19%), and > 20 sessions (8/91, 8.79%). Among them, 12 (13.19%) cases were of the nodular type, and 18 (19.78%) cases were of the hypertrophic type.

**TABLE 1:** Demographic data of all participants.

Basic Information	Options	Frequency	Percentage
Gender	Male	33	36.26
	Female	58	63.74
Age group (years)	< 3	31	34.07
	3–10	11	12.09
	11–20	18	19.78
	> 20	31	34.07
Fitzpatrick skin type	II	9	9.89
	III	22	24.18
	IV	60	65.93
Localization	Head and face	87	95.60
	Neck	3	3.30
	Trunk	2	2.20
	Extremities	2	2.20
Nodules	Yes	12	13.19
	No	79	86.81
Hypertrophies	Severe	0	0
	Moderate	1	1.10
	Mild	17	18.68
	No	73	80.22
Session of treatments	3–5	23	25.27
	6–10	28	30.77
	11–15	20	21.98
	16–20	12	13.19
	> 20	8	8.79

### 3.2 Main Conclusions

#### 3.2.1 Overall Analysis

**FIGURE 2:**
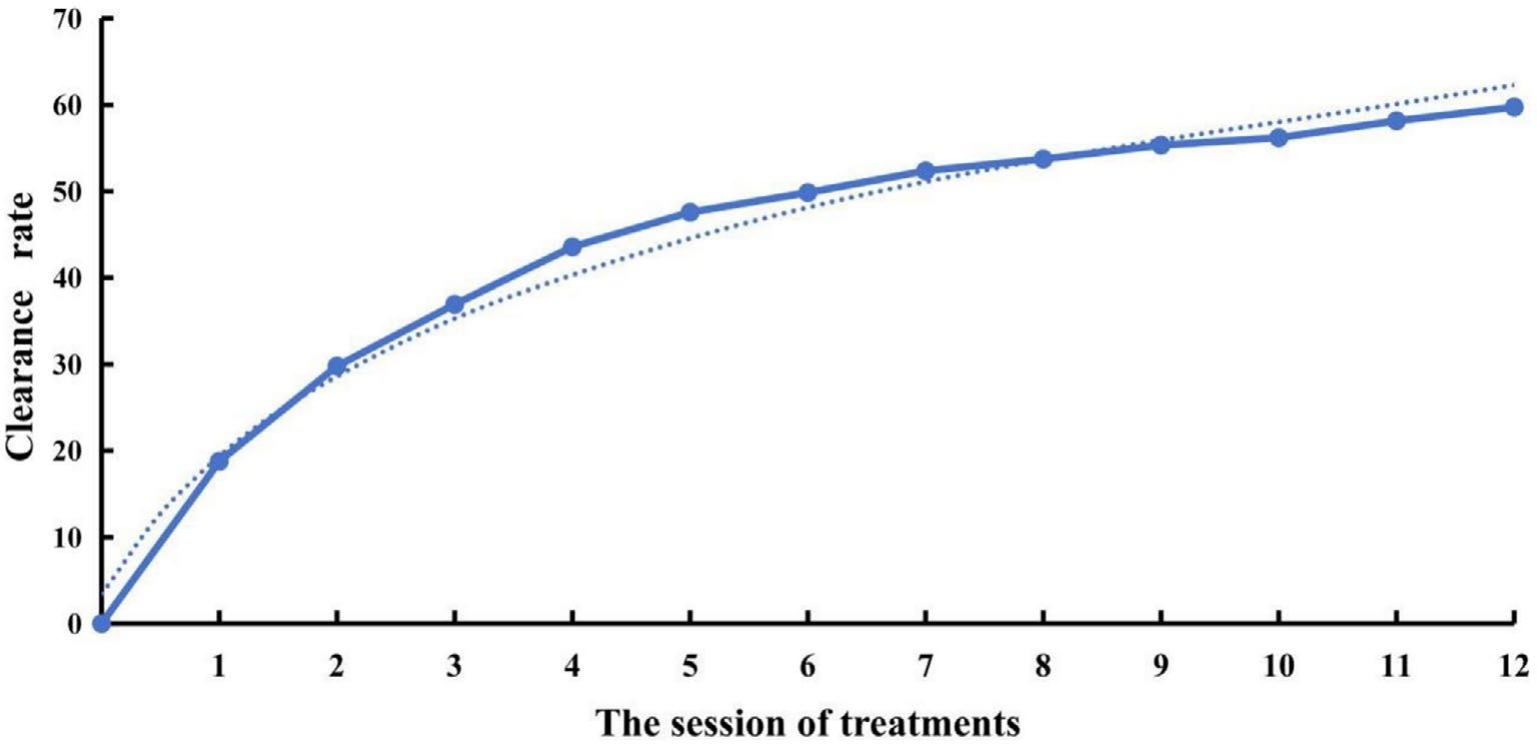
Clearance rate changing with the session of treatments.

To address missing values in the data, we employed multiple imputation to ensure data integrity and accuracy of analysis. We describe the mean change of clearance rate using mean and standard deviation (Figure 2). The results showed that the clearance rate increased gradually with increased sessions of treatment. Specifically, within the first 6 sessions of treatment, the mean change of clearance rate (49.85% ± 22.65%) was significant. Subsequently, they changed into a stable profile with the increasing sessions of treatment. The average clearance rate was 59.75% ± 18.86% under 12 sessions of treatment. The average session of treatments required to achieve a clearance rate of over 50% was 5.04 ± 3.17. After 4, 7, and 10 treatments, the proportions of patients achieving a clearance rate of ≥ 50% were 39.56%, 60.44%, and 68.13%, respectively. Meanwhile, the proportions of patients achieving a clearance rate of ≥ 75% were 15.38%, 13.19%, and 13.19%, respectively.

Adverse reactions of this study were slightly scaring (7/91, 7.69%). The patients with scarring all received more than 10 sessions of single PDL treatment, and fewer than four sessions of the combination treatment. Taken together, these results indicated that six sessions of combined treatment of PDL and CO_2_FL are necessary and can significantly improve the clinical manifestation in Chinese patients with PWS, with no obvious adverse reactions.

#### 3.2.2 Subgroup Analysis

**FIGURE 3:**
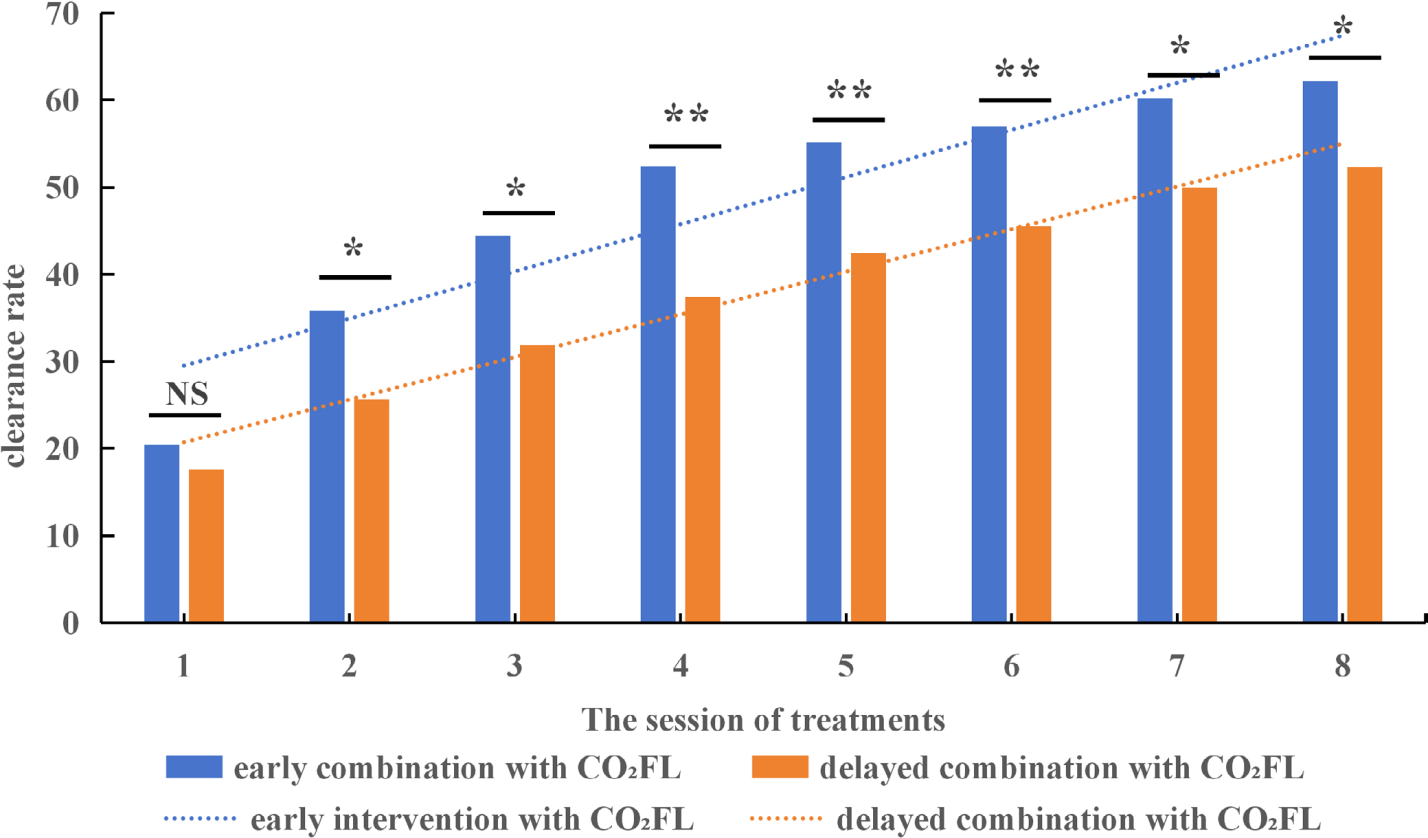
Comparison chart of clearance rates of the two groups of patients changing with the session of treatments. NS means *p* > 0.05, *means *p* < 0.05, **means *p* < 0.001.

**FIGURE 4:**
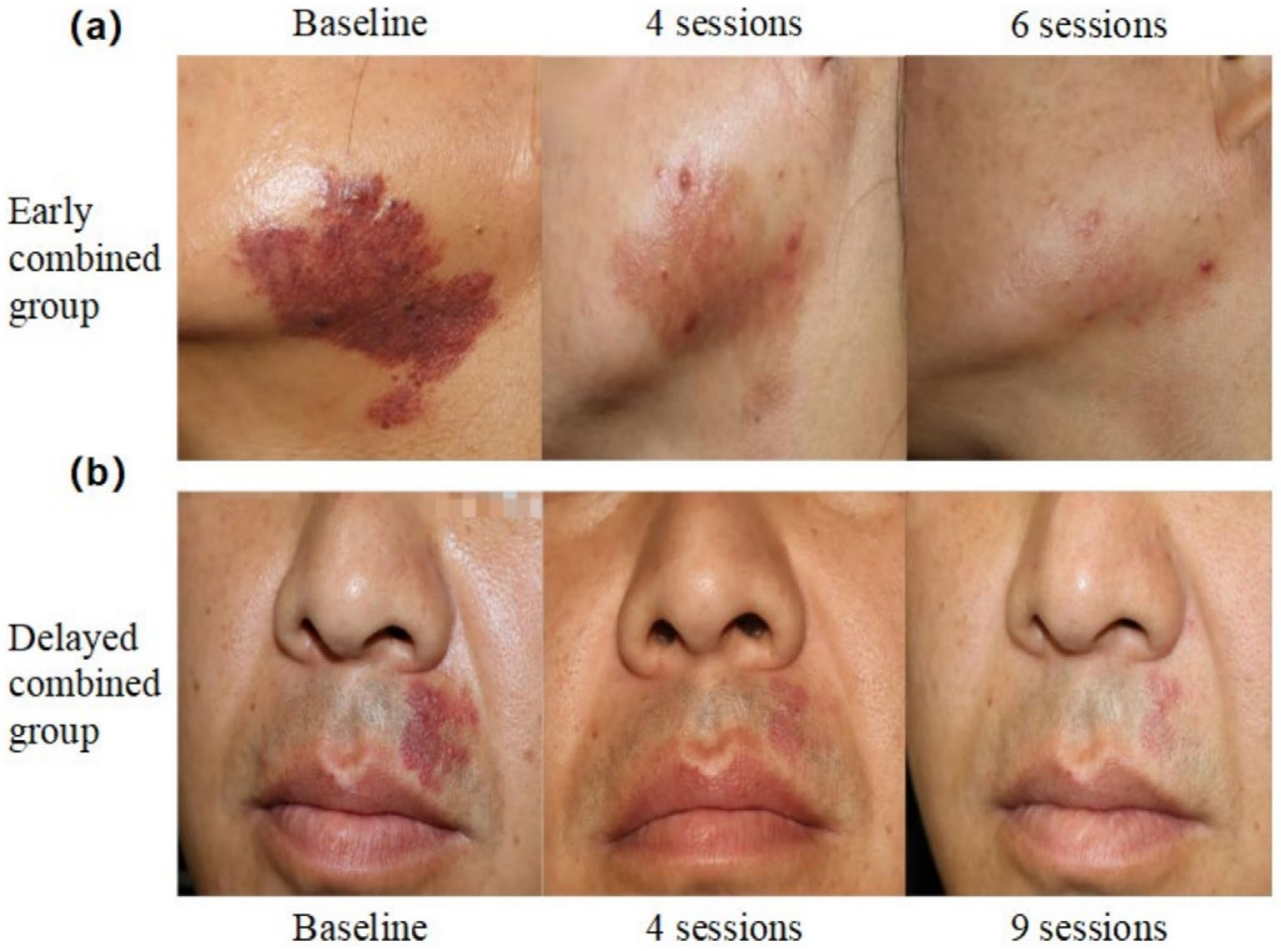
Clinical photographs demonstrated an enhancement in the appearance of PWS. (a) Photographs of a 52‐year‐old female with purplish‐red patches, mild hypertrophy and nodules at baseline. Lesion was improved after four sessions and six sessions of early combination of PDL and CO_2_FL treatments. (b) Photographs of a 36‐year‐old male with red patches at baseline. Lesion was improved after four sessions and nine sessions of delayed combination of PDL and CO_2_FL treatments.

To evaluate the optimal timing of the CO_2_FL combination, we categorized the patients into two groups: those who commenced the combination within the first five sessions, referred to as the early combination group (37/91, 40.66%), and those who began after five sessions, designated as the delayed combination group (54/91, 59.34%). The results showed that after eight sessions of treatment, the mean change of clearance rate was increased more significantly in the early combination group than the delayed combination group (62.23% ± 23.04% vs. 52.37% ± 22.23%, *p* = 0.003) (Table 2 and Figure 3). The clinical treatment outcomes of the two patients are presented in Figure 4, which also corroborates our research findings.

**TABLE 2:** Comparison of the differences in clearance rate between the two groups.

The session of treatments	Early combination with CO_2_FL		Delayed combination with CO_2_FL		OR (95% CI)	*p*
	Mean	SD	Mean	SD		
0	0.00	0.00	0.00	0.00	0.00 (0.00–0.00)	1.000
1	20.47	16.43	17.59	16.36	0.00 (0.00–0.00)	0.150
2	35.84	24.32	25.65	18.22	10.00 (0.00–10.00)	0.006
3	44.41	27.00	31.85	19.23	10.00 (0.00–20.00)	0.004
4	52.41	26.57	37.45	19.95	15.00 (8.92–23.00)	< 0.001
5	55.15	25.72	42.43	20.88	13.48 (6.26–21.43)	< 0.001
6	57.03	24.39	45.53	21.37	12.49 (5.00–20.72)	< 0.001
7	60.23	22.54	49.97	21.95	11.28 (4.46–19.58)	0.002
8	62.23	23.04	52.37	22.23	11.68 (3.79–19.04)	0.003

At the same time, we conducted an analysis of ordinal data between the two groups, and we respectively counted the proportions of patients in the two groups whose clearance rate reached ≥ 50% and ≥ 75% after four and eight sessions of treatment (Table 3). The results showed that after four sessions of treatment, the proportions of patients in the early combination group whose clearance rate reached ≥ 50% and ≥ 75% were 56.76% and 27.03%, respectively, which were significantly higher than those in the delayed combination group (27.78% and 5.56%, *p* = 0.005, *p* = 0.004). Consistently, after eight sessions of treatment, the proportions of patients in the early treatment group whose clearance rate reached ≥ 50% and ≥ 75% was higher than those in the delayed treatment group (89.19% vs. 35.14%, 59.26% vs. 14.81%, *p* = 0.002, *p* = 0.024).

**TABLE 3:** Comparison of ranked data between the two groups.

Clearance ratings	Early combination with CO_2_FL		Delayed combination with CO_2_FL		*p*	
	Session 4	Session 8	Session 4	Session 8	Session 4	Session 8
≥ 50%	21 (56.76%)	33 (89.19%)	15 (27.78%)	32 (59.26%)	0.005	0.002
≥ 75%	10 (27.03%)	13 (35.14%)	3 (5.56%)	8 (14.81%)	0.004	0.024

We also performed ordinal logistic regression analyses at session 4 and session 8. The results have been added to Table 4. At session 4, the treatment group was significantly associated with clearance level (*p* = 0.002). Compared with the delayed combination group, the early combination group was more likely to achieve a higher clearance category (OR = 3.83, 95% CI: 1.63–9.03). At session 8, the difference of clearance level between the treatment groups did not show statistical significance (OR = 1.97, 95% CI: 0.90–4.32, *p* = 0.090). The possible explanation is that patients in the delayed combination group had also initiated combination therapy by later treatment sessions, which may have improved their clearance outcomes and reduced the observed difference between the two groups. These results indicate that the early combination of PDL and CO_2_FL tends to exhibit more significant improvement than the delayed combination.

**TABLE 4:** Ordinal logistic regression analyses at session 4 and session 8.

Time point	Variable	*B*	OR (95% CI)	*p*	Model *p*
Session 4	Treatment group (early vs. delayed)	1.344	3.83 (1.63–9.03)	0.002	0.002
Session 8	Treatment group (early vs. delayed)	0.679	1.97 (0.90–4.32)	0.090	0.092

*Note:* OR = exp(*B*). Clearance levels were categorized as < 50%, 50%–75%, and ≥ 75%.

We performed multivariable analysis using the univariate general linear model, adjusting for age, lesion location, hypertrophic/nodular features, Fitzpatrick skin type, and number of treatment sessions. After adjustment, the clearance rate showed no difference between the treatment groups (tests of between‐subjects effects: *F* = 0.516, *p* = 0.475). Although parameter estimates suggested a lower adjusted clearance rate in the early combination group (*B* = −10.92), this difference was not statistically significant (*p* = 0.789, 95% CI: −91.905 to 70.066). These findings indicate that the differences observed in the univariate analysis may have been partially driven by baseline imbalances between groups. Furthermore, given that the delayed group included a higher proportion of very young patients (< 3 years), we conducted a sensitivity analysis excluding these patients and repeated the multivariable analysis. The results remained non‐significant (*p* = 0.379). The results suggest that early combination therapy is associated with better treatment outcomes, while emphasizing that this association may be influenced by residual confounding and does not imply causality.

#### 3.2.3 Multivariate Analysis for the Influencing Factors

In this section, we analyzed the potential influencing factors between the early combination group and the delayed combination group (Table 5). The early combination group included 37 patients, 11 male and 26 female; the delayed combination group included 54 patients, 22 males and 32 females. In terms of age distribution, patients with more than 20 years old accounted for the highest proportion in the early combination group (17/37, 45.95%), whereas patients with < 3 years old accounted for the highest proportion in the delayed combination group (22/54, 40.74%), which was a significant difference in age distribution between the two groups (*p* = 0.007). However, the early combination group has more nodules than the delayed combination group (9/12, 75% vs. 3/12, 25%, *p* = 0.009). In addition, in the early combination group, patients received 3–5 sessions of treatment, which accounted for the highest proportion (23/37, 62.16%); while patients received > 10 sessions accounted for the highest proportion (39/54, 72.22%) in the delayed combination group (*p* < 0.001). Taken together, compared with the delayed combination group, patients in the early combination group showed an older age, more nodular lesions, received fewer sessions of treatment, and achieved better effectiveness. In addition, we also present a comparative illustration of the clinical treatment outcomes for the two patients in Figure 4. These results further suggest that early combination of PDL and CO_2_FL tends to be a good choice for PWS treatment, especially better for the adult patients with nodules or hypertrophic lesions who are unsatisfied with PDL treatment.

**TABLE 5:** Analysis of the differences in influencing factors between the two groups.

Basic information	Option	Early combination with CO_2_FL		Delayed combination with CO_2_FL		*p*
		*F*	Percentage	*F*	Percentage	
Gender	Male	11	29.73	22	40.74	0.283
	Female	26	70.27	32	59.26	
Age group(years)	< 3	5	13.51	22	40.74	0.007
	3–10	7	18.92	8	14.81	
	10–20	8	21.62	10	18.52	
	> 20	17	45.95	14	25.93	
Localization	Head and face	36	97.30	51	94.44	0.515
	Neck	2	5.40	1	1.85	
	Trunk	0	0.00	2	3.70	
	Extremities	1	2.70	1	1.85	
Nodules	Yes	9	24.32	3	5.56	0.009
	No	28	75.68	51	94.44	
Hypertrophies	Yes	9	24.32	9	16.67	0.368
	No	28	75.68	45	83.33	
Session of treatments	3–5	23	62.16	0	0.00	< 0.001
	6–10	13	35.14	15	27.78	
	> 10	1	2.70	39	72.22	

Abbreviation: *F*, frequency.

## 4 Discussion

To our knowledge, this study included 91 PWS patients who received PDL combined with CO_2_FL treatment from January 2014 to December 2024. This study is the first to systematically analyze the effectiveness of PDL combined with CO_2_FL treatment for PWS in a relatively large sample size. Moreover, we found that early combination with CO2FL may be associated with better clinical benefits to patients. This finding provides new ideas and insights for the clinical treatment of PWS.

Pulsed dye laser (PDL) is recognized as the gold standard for treating PWS, but complete PWS clearance is rarely achieved. Most patients require 8–10 sessions of treatment or more for optimal results. PDL can achieve 50%–90% clearance, and the majority of patients will have approximately 50% lightening [18]. 35% of patients experienced a recurrence of lesion color after PDL treatment [19]. Thus, combination with PDL has been explored, including combination with angiogenesis inhibitors, such as rapamycin and imiquimod, or combination with other laser technologies, such as longer wavelengths (755 nm, 1064 nm), intense pulsed light, or photodynamic therapy. These devices may help target larger or deeper vessels, such as patients with nodular and hypertrophic lesions; however, they have a higher risk of damage to non‐targeted tissue than PDL, which may increase the risk factor of adverse effects, such as hyperpigmentation, atrophy, and scarring.

Ablative fractional laser (AFL) can make rapid heating and vaporization of skin tissue to the deep dermis in the pattern of fractional photothermy. When hypertrophic scar or immature scar was treated using AFL, the erythema of the scar can also lighten [20], which indicates that AFL may be a potential method to treat PWS by vaporization of vessels in the dermis. Zhang [14] reported two PWS cases treated with CO_2_FL. The results showed that compared with conventional treatment, both patients achieved better therapeutic effects, with reduced risks of adverse reactions such as infection, pigmentation, and scar formation, decreased clinical erythema, and significantly reduced pain. Toren and Marquart [15] reported three PWS patients with PDL‐resistance treated with a combination of PDL and Er: YAG fractionated laser.

In our study, considering CO_2_FL can induce deeper and wider tissue vaporization and thermal injury, we performed CO_2_FL combined with PDL to treat PWS patients. This prospective study included 91 patients, and the results showed that the average clearance rate was 59.75% ± 18.86% under 12 sessions of treatment. The average session of treatments required to achieve a clearance rate of over 50% was 5.04 ± 3.17. After 4, 7, and 10 treatments, the proportions of patients achieving a clearance rate of over 50% were 39.56%, 60.44%, and 68.13%, respectively. Meanwhile, the proportions of patients achieving a clearance rate of over 75% were 15.38%, 13.19%, and 13.19%, respectively. Our research findings above are consistent with previous studies in Asian populations [21]. This study analyzed the timing of the combination of PDL and CO_2_FL. The results show that the early combination group tend to be superior to the delayed combination group. As we know, the effectiveness of PDL is influenced by various factors, such as lesion location, vascular characteristics, age, Fitzpatrick skin type [2], and et al. Patients with lighter skin types have a better treatment response. PWS on the lateral face respond better than those in the central face. Pink, red, and reticular lesions respond better than those that are purple and geographically shaped. PWS with hypertrophic character show a poorer response than those that are flat and smooth. We also analyzed the differences in influencing factors between the two groups and found that patients in the early combination group showed older age, more nodular lesions, received fewer sessions of treatment, but showed better effectiveness, compared with the delayed combination group. Thus, early combination of PDL and CO_2_FL is associated with better benefits for adult PWS patients, especially those with nodular hypertrophy lesions.

Previous studies have found that repeated PDL treatment can lead to post‐inflammatory hyperpigmentation, skin atrophy, and scar formation, with an increased incidence of adverse reactions as the number of treatments increases [22]. CO_2_FL can not only prevent scar formation but also has a good therapeutic effect on existing scars and skin atrophy. Our results also found that 7/91 (7.69%) patients have slight scarring, and these patients received more than 10 sessions of single PDL treatments and less than four sessions of combination treatment. Previous studies have shown that the incidence of scarring and atrophy was positively associated with the increasing number of sessions of PDL treatment [22]. Thus, we consider that the scaring in the study may have been caused by multiple sessions of single PDL treatment, but not the combination treatment.

## 5 Limitations

Although our study has to some extent demonstrated the effectiveness of a combination of PDL and CO_2_FL in treating PWS, there are certain limitations. This is a retrospective study; the integrity and accuracy of the data may be restricted. The influence of unavoidable confounding factors and selection bias may also interfere with the true assessment of the treatment effect. We also emphasize that this association should be interpreted cautiously because of the potential for insufficient statistical power, residual confounding, and selection bias inherent to the retrospective design. Therefore, prospective randomized controlled trials are needed to further verify the effect of combined treatment in PWS.

## 6 Conclusion

The results of this study indicate that the combination of PDL and CO_2_FL is an effective and promising option for PWS treatment. The timing of the intervention significantly impacts the treatment outcome. Specifically, the early combination group tend to get significantly better outcomes in terms of clearance rate compared with the delayed combination group, demonstrating that introducing the combined treatment with CO_2_FL in the early phase is associated with better treatment outcomes.

## Author Contributions

Bing Li and Lin Gao were responsible for the study design. Jine Zhang and Pu Song were responsible for evaluating the treatment effects of patients before and after the therapy. Mengjiao Li, Yang Yang, Xiaolei Su, Caiyu Wang, Ping Jian were in charge of gathering the aforementioned assessment data as well as the patients' basic information, such as photos. Sample size calculation and statistical analysis were performed by Jiao Ning and Guotao Fu. Jiao Ning wrote the manuscript. All authors contributed to the data interpretation, critically reviewed the manuscript and approved the submitted version.

## Funding

This work was supported by the National Natural Science Foundation of China, 82073435.

## Ethics Statement

This study was a retrospective observational study, and approved by the Ethics Committee of Air Force Medical University in China (Ethics no. KY20242205).

## Consent

All participants provided their written informed consent.

## Conflicts of Interest

The authors declare no conflicts of interest.

## Data Availability

The data that support the findings of this study are available from the corresponding author upon reasonable request.
